# AGE DIFFERENCES IN PSYCHOLOGICAL DISTRESS AFTER MULTIPLE DISASTER EXPOSURES: EFFECT OF NEGATIVE COVID-19 IMPACTS

**DOI:** 10.1093/geroni/igad104.2661

**Published:** 2023-12-21

**Authors:** Zhirui Chen, Zhen Cong

**Affiliations:** The University of Alabama at Birmingham, Birmingham, Alabama, United States; The University of Alabama at Birmingham, Birmingham, Alabama, United States

## Abstract

This study examined how older adults differed from their younger counterparts in psychological distress following exposures to tornadoes and the COVID-19 pandemic; and how the multidimensional negative COVID-19 impacts contextualized the above age differences. Data used were from a two-wave panel study of tornado victims during the COVID-19 pandemic (N = 554). Latent class analysis was first conducted to explore the underlying patterns of negative COVID-19 impacts at T1 (October 2020-August 2021). Negative binomial regressions were performed to examine the age differences in psychological distress at T2 (May-August 2022), as well as the moderating effect of the identified latent classes, with baseline psychological distress and covariates controlled. Three latent classes were identified: class 1 “low overall impacts” (39.24%), class 2 “moderate overall impacts with high emotional distress” (47.71%), and class 3 “severe overall impacts” (13.05%). Individuals aged 65+ reported lower psychological distress at T1 relative to those aged 18-34, 35-49, and 50-64, and their advantages in mental health over people under 50 can be maintained over time. However, compared to people aged 18-34, 35-49, and 50-64, those aged 65+ reported the greatest increases in T2 psychological distress if they had experienced moderate or severe overall COVID-19 impacts at T1. These findings highlighted the resilience and vulnerability of older adults in post-disaster recovery. As the frequency and intensity of cumulative disasters increase across the globe, there is a pressing need for mental health interventions that are tailored to multi-disaster scenarios and age-related differences in long-term disaster recovery.

